# Integrating climate change and health topics into the medical curriculum – a quantitative needs assessment of medical students at Heidelberg University in Germany

**DOI:** 10.3205/zma001618

**Published:** 2023-05-15

**Authors:** Leonie Rybol, Jessica Nieder, Dorothee Amelung, Hafsah Hachad, Rainer Sauerborn, Anneliese Depoux, Alina Herrmann

**Affiliations:** 1 University Hospital Heidelberg, Heidelberg Institute of Global Health, Heidelberg, Germany; 2 University Heidelberg, Medical Faculty, Heidelberg, Germany; 3 Sorbonne University, Medical Faculty, Paris, France; 4 University of Paris Cité, Centre Virchow-Villermé, Paris, France; 5 University Hospital Cologne, Medical Faculty Cologne University, Institute for General Medicine, Cologne, Germany

**Keywords:** climate change, health, knowledge, medical curriculum, students' needs assessment

## Abstract

**Objectives:**

Climate change (CC) is of major importance for physicians as they are directly confronted with changing disease patterns, work in a greenhouse gas intensive sector and can be potential advocates for healthy people on a healthy planet.

**Methods:**

We assessed third to fifth year medical students’ needs to support the integration of CC topics into medical curricula. A questionnaire with 54 single choice-based items was newly designed with the following sections: role perception, knowledge test, learning needs, preference of educational strategies and demographic characteristics. It was administered online to students at Heidelberg medical faculty. Data sets were used for descriptive statistics and regression modelling.

**Results:**

72.4% of students (N=170, 56.2% female, 76% aged 20-24 years) (strongly) agreed that physicians carry a responsibility to address CC in their work setting while only 4.7% (strongly) agreed that their current medical training had given them enough skills to do so. Knowledge was high in the area of CC, health impacts of CC, vulnerabilities and adaptation (70.1% correct answers). Knowledge gaps were greatest for health co-benefits and climate-friendly healthcare (55.5% and 16.7% of correct answers, respectively). 79.4% wanted to see CC and health included in the medical curriculum with a preference for integration into existing mandatory courses. A multilinear regression model with factors age, gender, semester, aspired work setting, political leaning, role perception and knowledge explained 45.9% of variance for learning needs.

**Conclusion:**

The presented results encourage the integration of CC and health topics including health co-benefits and climate-friendly healthcare, as well as respective professional role development into existing mandatory courses of the medical curriculum.

## Zusammenfassung

**Zielsetzungen:**

Der Klimawandel ist für Ärzt*innen von großer Bedeutung, da sie mit sich verändernden Krankheitsbildern konfrontiert sind, in einem treibhausgasintensiven Sektor arbeiten und potenzielle Fürsprecher für gesunde Menschen auf einem gesunden Planeten sind.

**Methoden:**

Wir untersuchten die Lernbedürfnisse der Medizinstudierenden im dritten bis fünften Studienjahr im Bezug auf die Integration von Klimawandel und Gesundheits-Themen in die medizinischen Lehrpläne. Es wurde ein neuer Fragebogen mit 54 Items entwickelt. Dieser enthält die folgenden Abschnitte: Rollenwahrnehmung, Wissen, Lernbedürfnisse, Präferenz von Lernstrategien und demografische Merkmale. Der Fragebogen wurde online an Studierende der Medizinischen Fakultät Heidelberg verschickt. Die Datensätze wurden mittels deskriptiver Statistik und Regressionsanalyse ausgewertet.

**Ergebnisse:**

72,4% der Studierenden (n=170, 56,2% weiblich, 76% im Alter von 20-24 Jahren) stimmten (stark) zu, dass Ärzt*innen Verantwortung dafür tragen, den Klimawandel in ihrem Arbeitsumfeld zu adressieren. Nur 4,7% stimmten (stark) zu, dass in ihrer bisherigen medizinischen Ausbildung genügend Fähigkeiten und Wissen vermittelt wurden, um dies zu tun. Der Wissensstand war in den Bereichen Klimawandel, gesundheitliche Auswirkungen, Vulnerabilität und Anpassung hoch (70,1% richtige Antworten). Die größten Wissenslücken gab es bei den gesundheitlichen Zusatznutzen („Health Co-Benefits“) und der nachhaltigen Gesundheitsversorgung (55,5% bzw. 16,7% richtige Antworten). 79,4% der Befragten wünschten sich, dass die Themen im Bereich Klimawandel und Gesundheit in das medizinische Curriculum aufgenommen werden („Lernbedürfnis“), wobei sie eine Integration in bestehende Pflichtkurse bevorzugten. Ein multiples lineares Regressionsmodell mit den Faktoren Alter, Geschlecht, Semester, angestrebtes Arbeitsumfeld, politische Orientierung, Rollenwahrnehmung und Wissen erklärte 45,9% der Varianz des Lernbedürfnisses.

**Schlussfolgerung:**

Die vorgestellten Ergebnisse ermutigen dazu, Themen zu Klimawandel und Gesundheit, in bestehende Pflichtkurse des medizinischen Curriculums zu integrieren. Dabei sollten auch gesundheitliche Co-Benefits, eine klimafreundliche Gesundheitsversorgung und die Entwicklung eines entsprechenden ärztlichen Rollenverständnisses berücksichtigt werden.

## 1. Introduction

Climate change (CC) is an increasing threat to human health. The World Health Organization predicts 250 000 additional deaths per year attributable to CC by 2030 if no further action is implemented [1]. At the same time, CC mitigation can benefit human health [2], [3]. Hamilton et al. [4] showed that CC mitigation measures in nine countries around the world could lead to an annual reduction of about eight million deaths by 2040 due to lower risks of air pollution, poor diet, and low physical activity levels. Within that space of risks and opportunities, medical doctors hold a key role. They are confronted with changing disease patterns due to CC and need to adapt their clinical care accordingly. Furthermore, physicians can help to deliver sustainable health care [5] and play an important advocacy role, either by counselling their patients on climate change and health [6], [7] or by encouraging climate action in society at large [8]. 

Therefore, the Association for Medical Education in Europe (AMEE) and others call for an integration of CC, planetary health and sustainable health care into medical curricula [9], [10], [11], [12], [13], [14], [15], [16], [17]. Yet in 2020, Omrani et al. [18] showed that of 2817 surveyed medical schools worldwide, only 15% addressed Climate Change and Health (CC&H) in their curricula. In Germany, the Institute for Medical and Pharmaceutical Exam Questions (IMPP) is now developing a blueprint for the subject catalogue for planetary health and CC to be integrated into medical examinations [19], which further emphasizes the need for curriculum adjustments to include CC&H subjects. 

According to Thomas et al. [20], the first step of curriculum development is “performing a needs assessment”. While an external need is evident, the needs of the learners (i.e., medical students) are not as established. Schneiderhahn et al. [21] particularly point to the assessment of attitudinal and knowledge-based needs as well as readiness to learn and preferences for educational strategies. With regard to attitudinal needs, data from China, Ethiopia, India and the USA show that medical students are mostly aware of the health hazards stemming from CC and realize that as physicians they have a role to play in CC. At the same time, they do not feel prepared for these challenges [22], [23], [24], [25], [26], [27]. With regard to knowledge-based needs, current studies mostly focus on general knowledge about CC and its health impacts, but rarely consider vulnerability, co-benefits or climate-friendly healthcare. Thus, comprehensive knowledge-based needs assessments and assessments of preferences for teaching strategies for medical students are lacking, particularly in Europe. 

We therefore conducted a survey study with third to fifth year students at Heidelberg Medical Faculty to answer the following research questions:

1. What are medical students’ individual and professional role perceptions in CC&H?
2. What is their knowledge with regard to CC, health impacts, adaptation, vulnerabilities, health co-benefits and a climate-friendly healthcare?
3. What are their perceived learning needs and their preference for educational strategies towards inclusion of CC and health topics into medical curricula?
4. What factors (demographic, role perception, knowledge) explain their learning needs?

## 2. Materials and methods

### 2.1. Study design 

We conducted a cross-sectional survey as a self-administered online questionnaire (via Lime Survey Version 5.3.22), which was completed anonymously.

### 2.2. Study setting and recruitment

The study was conducted among medical students at the medical faculty of the University of Heidelberg. Medical school in Germany is divided into a two-year pre-clinical phase, and a four-year clinical phase. At the time of the survey, CC education was not an obligatory part of the curriculum in Heidelberg.

We invited all students from three different courses (unrelated to CC&H topics) in the clinical phase (third to fifth year) of Heidelberg medical school in 2021 and 2022 (788 students in total). We limited the distribution of the survey to three courses only to focus our recruitment efforts and maximize the response rate: in two courses, students were invited to participate at in-person or online lectures and reminded via e-mail as well as on the faculty’s online platform and social media. In the third course, or they were approached at the end of an online lecture to complete the survey within the scheduled lecture time.

### 2.3. Survey design 

We assessed the role perception, medical knowledge, learning needs and preferences for educational strategies alongside demographic characteristics (age, gender, semester, aspired specialty and career and political leaning) of medical students in the area of CC&H by use of a newly developed questionnaire instrument. For role perception, learning needs and preferences for educational strategies we used a five-point Likert scale, while students could one out of three answers (correct, incorrect, do not know) in the knowledge section. Our multidisciplinary research team developed the survey items in an iterative process, with 19 of the 54 items adapted from previously published questionnaires (see attachment 1 ). As survey items were originally developed in English, they were translated into German and back-translated into English independently by two bilingual colleagues to ensure accuracy. 

A pilot test on four medical students was conducted to check understanding of all items and minor adjustments in formulation of items was performed. 

#### 2.3.1. Survey sections 

##### Role perception

We used the constructs of individual and professional role perception to assess attitudinal needs. We included two self-developed items on personal responsibility (R1-2) and three adopted items on professional responsibility (R3-5) [28], [29].

##### Knowledge-based needs

For the knowledge-based needs section, we screened the existing literature on CC&H curricula for potential themes [9], [10], [30], [31], [32], [14], [33], [34], [17] and prioritized the following:

1. *CC in general, *
2. *health impacts of CC, *
3. *CC vulnerability, *
4. *adaptation to health impacts of CC *
5. *health co-benefits of climate action *
6. *climate-friendly healthcare. *

Each knowledge domain was made up of five statements, which students could rate as “correct”, “incorrect” or “do not know”.

##### Learning needs 

In addition to a general assessment of students’ perceived learning needs with regards to CC&H topics within the medical curriculum (L1: “*In your opinion, should teaching about climate change and health be integrated into the medical curriculum?*”), we also asked for the learning needs in five key domains (L2-6). 

##### Preferences for educational strategies

We included five items on the preferences fordifferent strategies for integration into the medial curriculum (e.g., integration into existing mandatory courses) (L7-11). 

### 2.4. Data analysis 

Only fully completed questionnaires were included in the analysis. Basic descriptive statistics were conducted on all possible variables to describe the characteristics of the study group. Repeated measures ANOVAs with Greenhouse-Geiser correction were used to investigate differences in correct responses and educational preferences. To understand what factors might be relevant in shaping medical students' learning needs, pairwise correlation analyses and multiple linear regression analysis were performed. A p-value <0.05 was considered statistically significant. 

### 2.5. Ethical issues 

This study was conducted in line with the declaration of Helsinki from 2019 as well as the General Data Protection Regulations. All students gave informed consent to the online survey before participating. The study protocol was approved by the ethics committee of the Heidelberg Medical Faculty (S-428/2021).

## 3. Results

### 3.1. Sample description 

In total, 214 responses were collected from 788 potential respondents (27.03% response rate), with 170 fully completed surveys (adjusted response rate 21.5%). A majority of students was female (56.21%), in their propaedeutic semester (third year; 64.12%) and voted for the green party (58.04%) (see attachment 1 , here table S6).

### 3.2. Internal consistency

In order to measure internal consistency of our newly developed instrument we calculated Cronbach’s Alpha for each scale (see attachment 1 , here table S7). Results indicated sufficient reliability ranging from α=0.86 (learning needs) over α=0.81 (knowledge) to α=0.71 (role perception).

### 3.3. Role perception 

**Figure 1:**
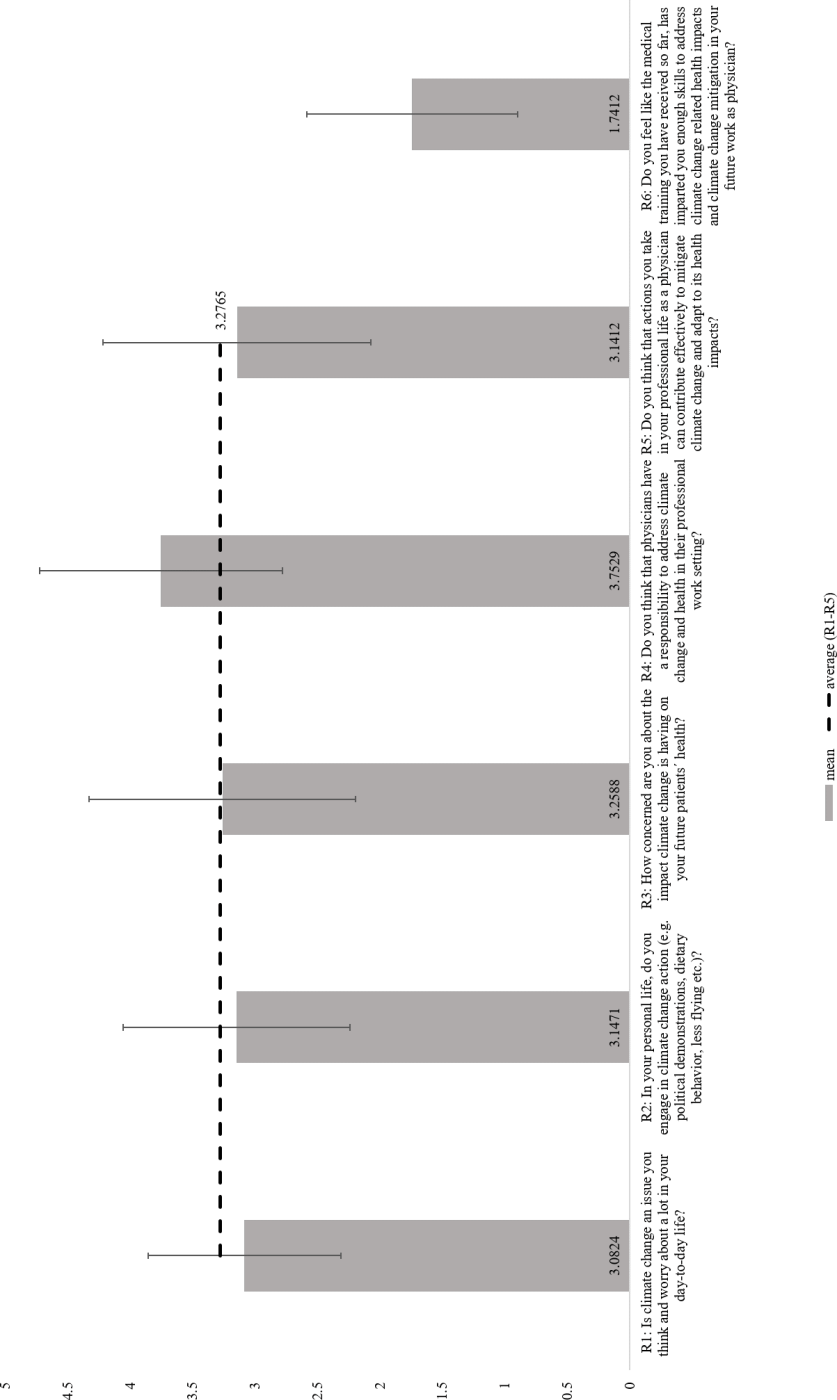
Role perceptions. Ranges show standard deviations from mean

72.35% of medical students agreed or strongly agreed that “*physicians have a responsibility to address CC&H in their professional work setting”*. Significantly fewer students agreed that “*actions they take in their professional life as a physician can contribute effectively to mitigate CC and adapt to its health impacts*” (42.95%). Only 4.71% agreed or strongly agreed, that “*the medical training they had received so far, had imparted them with enough skills to address CC related health impacts and CC mitigation in their future work as physicians*” (see attachment 1 , here table S1). The results of role perceptions are displayed in figure 1 (Fig. 1). 

### 3.4. Knowledge-based needs

On average, students correctly answered 18 out of 30 (SD=4.49; range 3-20), and incorrectly answered two out of 30 knowledge items (SD=1.66, range 0-7). On average, ten out of 30 statements were marked as “do not know” (SD=5.08, range 0-27) (see attachment 1 , here table S3). With item difficulties ranging from 3% to 100% correctly answered questions we find that the scale overall did not appear to be too easy or hard for our sample. Variability in the distribution of correct answers was comparable across knowledge categories in the survey (see attachment 1 , here table S2, figure S1 and figure S2). 

**Figure 2:**
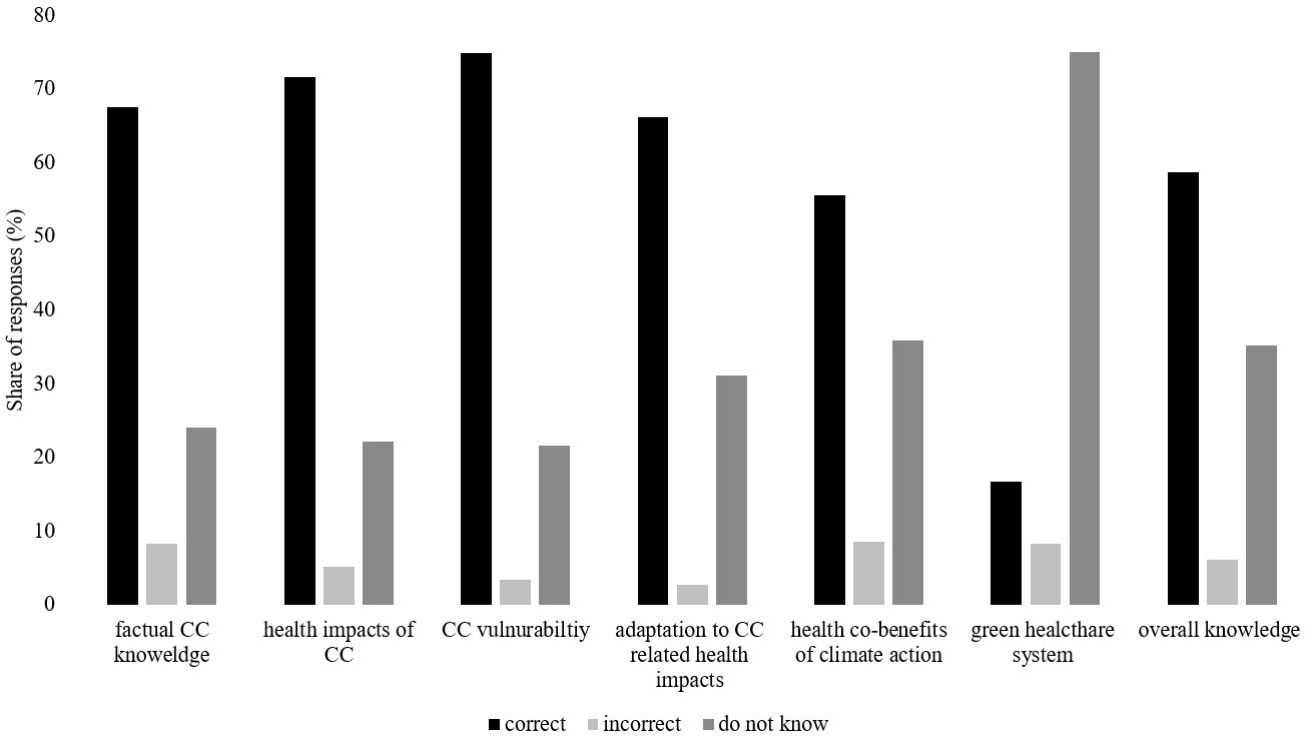
Correctly and incorrectly answered items per knowledge category and in total

Students’ knowledge differed significantly between categories (F(4.586, 775.024)=207.089, p<0.001) (see figure 2 (Fig. 2)). Post hoc analyses with Bonferroni adjustment showed that the share of correctly rated statements was highest for *CC knowledge* (68%), *health impacts of CC* (71.65%), *CC vulnerability* (74.96%) and* adaptation to CC related health impacts* (66.24%). *Co-benefits of climate action* had significantly lower rates of correct answers (55.29%, p=0.002), whilst knowledge gaps were most prominent in the area of *climate-friendly healthcare* (17%, p<0.001). Notably, the occurrence of incorrect responses was not significantly higher in this category (8.24%) than within *CC knowledge* (8%, p=1), rather there was a higher frequency of “do not know” responses than in all other categories (35.16%, p<0.001). For example, 82.35% indicated not knowing whether the statement “*In European countries greenhouse gas emissions form the health sector represents approximately 5% of all national greenhouse gas emissions*” was correct or incorrect. Response statistics to each item can be reviewed in attachment 1 , here table S2. 

### 3.5. Learning needs and preferences for educational strategies 

**Figure 3:**
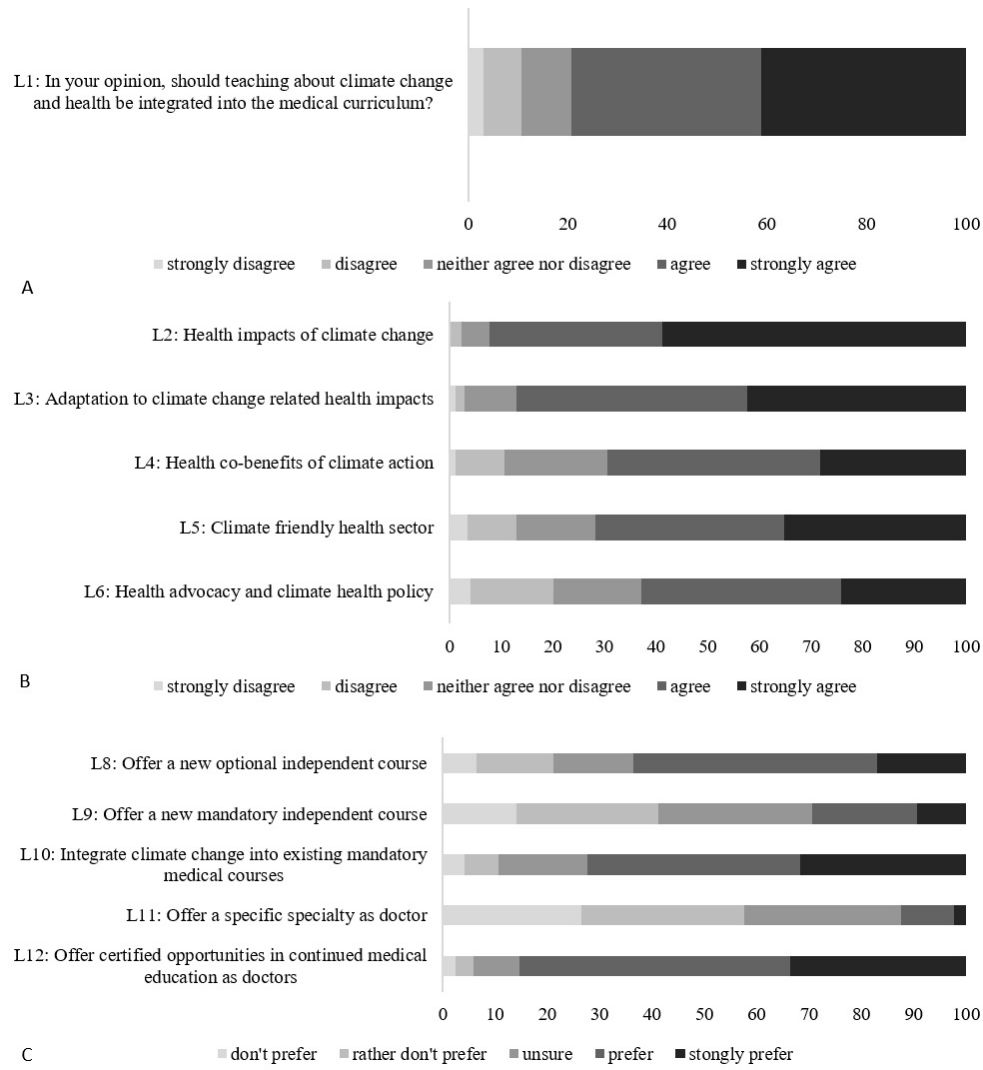
A: Learning need. B: Topical preferences. C: Teaching preferences indicated by students. Frequencies are shown in % based on 170 responses.

79.4% of the students agreed or strongly agreed, that teaching about CC&H should be integrated into the medical curriculum (see figure 3 (Fig. 3)). 

When asked about specific topics, most students (strongly) agreed (92.4%) that health impacts of CC should be included, this was followed by adaptations to CC related health impacts (87.06%, p<0.001), followed by climate-friendly healthcare (71.76%, p<0.001) and health co-benefits (69.42%, p<0.001). Students were least interested in the topic of health advocacy and climate policy (69.24%, p=0.004). A post-hoc analysis with Bonferroni adjustment of a repeated measures ANOVA showed that preferential differences were significant (F(3.297, 557.268)=40.201, p<0.001).

With regard to the preference of educational strategies, it is notable, that certified continued medical education courses (85.29%) and the integration into existing mandatory courses (72.34%) were significantly preferred over other options, such as voluntary electives or separate obligatory courses. Specific results on both sections are depicted in figure 3 (Fig. 3) and detailed in attachment 1 , here table S5.

### 3.6. Multiple linear regression model for learning needs 

We conducted an exploratory regression analysis with age, gender, semester, aspired work setting, political leaning, role perception and knowledge as predictors. We found that the model significantly predicted learning needs (Score L1-L6, Cronbach’s alpha 0.855) explaining 45.9% of variance (F(7, 132)=17.86, p<0.001) (see attachment 1 , here table S9). Gender, semester, and role perception significantly added to the prediction. Specifically, students identifying as female, in a higher semester and with a stronger role perception expressed higher learning needs. 

## 4. Discussion

The aim of this study was to assess Heidelberg medical students’ needs for the integration of CC&H into the medical curriculum. We found that students felt responsible to address CC&H in a professional setting, but had some relevant knowledge gaps, particularly with regard to co-benefits of climate action and climate-friendly healthcare. Students were ready to learn about CC&H, specifically about health impacts and adaptation, while they were not as interested in health advocacy and climate health policy. They preferred the integration of CC topics into existing mandatory courses of their curriculum and their learning needs were strongly associated with role perception. 

### 4.1. Role perception

76% of medical students in our sample felt worried about CC personally and 73% indicated being engaged in climate action. While we could not find a comparable survey question with medical students, Kotcher et al. [13] found a similar rate of concern amongst physicians about CC and their patients' health. It is important to note that a majority of students in our sample felt that physicians have a responsibility to address CC&H in their professional work setting and they had a rather strong feeling of self-efficacy as doctors in CC. However, perceived self-efficacy was significantly lower than their perceived responsibility. Other international surveys with health professionals mainly support this finding [13], [29], stressing the fact that medical students need to learn how to act effectively on CC. 

Bugaj et al. [35] found that agreement to a professional responsibility in CC was significantly lower than to a personal responsibility. At first sight, these findings seem to contradict the results of this study. However, the items measuring professional responsibility in Bugaj’s work differed from ours. While their wording pertained to a social role model and educational function of physicians, ours emphasised physicians’ role of adapting to and mitigating CC in the healthcare setting. 

### 4.2. Knowledge-based needs 

Students in this survey had a good metacognition of what they knew or did not know about CC&H. Overall, knowledge on CC and the health impacts of CC was relatively high among students at Heidelberg medical faculty with knowledge scores similar to those found in other international studies [24], [25]. At the same time, knowledge on climate-friendly health care was low, which also corresponds to findings of Ryan et al. [26], which showed that medical students had little knowledge on emissions of health care. However, as knowledge was assessed differently across surveys, a direct comparison is difficult. In line with our study, research from China, Ethiopia, and the USA, found that medical students do not feel that their current medical training has adequately prepared them to address CC in their future work [22], [23], [24], [25], [27].

### 4.3. Learning needs and preferences for educational strategies

Earlier we have found that a professional role emphasizing physicians' role of adapting to and mitigating CC in the healthcare setting seems to be more acceptable than a professional role stressing the social and educational function of physicians. If we take into account, that medical students in our survey were more eager to learn about health impacts of CC and adaptation than about health advocacy and climate health policy, this might suggest that medical students do perceive a responsibility in the direct healthcare setting, but not in a wider societal advocacy role. This is further supported by findings from Liao et al. [23] indicating that medical students want to learn about CC related clinical knowledge and skills, but less about legal and ethical frameworks. Nonetheless, most experts on the curriculum development of CC&H support the integration of leadership and advocacy including “communication of sustainability values” [15], [36]. This suggests that medical students have not yet embraced the full leadership role in CC that health professionals could adopt [15], [36]. Therefore, role perception and leadership should be addressed in curriculum development, although or even because it does not currently seem to be the top priority of medical students. 

With regard to preferences for educational strategies, it was striking that our study population preferred the integration of the topic into existing mandatory courses. This stands in contrast to current practice in German medical schools, which – if at all – offer CC&H electives. It also supports the recommendation of the AMEE statement to “mainstream planetary health as a transversal curricular theme” [15]. It was projected, that integrating CC topics into existing courses at an American medical faculty would only marginally increase classroom time [14]. Therefore, this integration might even be more feasible in already packed medical curricula with rivalling opportunities. However, this implies, that competencies with regard to CC&H need to be built among medical school teachers of all specialities. Students wanting CC&H education introduced into continued medical education courses for trained doctors supports claims that CC should be integrated on all levels of the medical education system.

### 4.4. Multiple linear regression model for learning needs

One predictor for learning needs was female sex. This is in line with the general finding in studies about predictors of pro-environmental and climate-friendly behaviour, according to which women express higher rates of environmental concern and a higher readiness to act upon this concern [37], [38]. Earlier in the discussion we concluded, that role perception and leadership should be part of the curriculum development. In the regression modelling we found, that stronger role perception was associated with a greater learning need for CC&H, whereas better knowledge on CC topics did not directly affect learning needs. This further strengthens the point, that professional role development is important to educate medical students holistically about CC&H.

### 4.5. Strengths and limitations of the study 

To our knowledge, our study is the first in-depth assessment of knowledge-based needs of medical students with regard to CC. Particularly, we do not know of other studies assessing medical students’ knowledge about adaptation to health impacts of CC and health co-benefits of climate action. 

A relevant limitation is the response rate of 27.16% giving way to selection bias. The response rate is similar to other surveys in this field, mostly ranging between 10-30% [39], [22], [13], [29], with some outliers of 87% and 97% [35], [23]. To get an idea of the extent of the bias, we compared the sample demographics with other population demographics. Our age range mostly reflected the age of medical students in Germany, who start their medical studies around 18 years and finish at 26 [40]. The share of 56% of female students in our sample is lower than the German medical student average of 64% in 2020/2021 [41]. It is striking, that 58% of respondents indicated voting for the green party. We were unable to find representative voting behaviour of German medical students, yet only 23% of all German voters aged 18-24 voted for the green party in the last election [42]. This suggests a non-response bias meaning that students voting for parties other than the green party were less likely to participate in the survey which could suggest an overestimation of role perception, knowledge and educational preference in our sample compared to the full population. Yet, with regard to the knowledge gaps in the area of health co-benefits and climate-friendly healthcare, this potential bias is unlikely to make a relevant difference at least with regard to knowledge. Finally, our sample was restricted to students from only one medical faculty in Germany, which could limit generalizability of the results. Yet, as outlined in the discussion, our findings resonate well with findings from other medical student populations globally, suggesting that our results could also hold for other medical student populations.

## 5. Conclusion

The presented study corroborates findings of previous studies, that medical students do perceive a responsibility in addressing CC as future doctors. Furthermore, results point at health impacts and adaptation as greatest areas of interest for medical students and at health co-benefits and climate-friendly healthcare as areas with lowest knowledge and lowest perceived learning needs. Our findings encourage the integration of CC topics, including health co-benefits and climate-friendlyhealthcare, into existing mandatory courses of the medical curriculum. Furthermore, our results underline the claim, that professional role development should be one integral part of such curricula. 

## Acknowledgements

We are thanking all students participating in the survey and staff at the medical faculty of the university of Heidelberg, who supported data collection. 

## Authors

- Leonie Rybol has been a medical student at Heidelberg University since 2017. For her doctoral thesis on climate change in medical education, she is working at the Heidelberg Institute of Global Health.
- Jessica Nieder is a PhD student at the Heidelberg Institute of Global Health. With a background in Health Psychology, she studies digital education for climate change and health.
- Dorothee Amelung is a researcher with a background in psychology, and an interest in sustainable behavior change. Dorothee has held a post doc position at University of Surrey, UK, and currently holds a post doc position at Heidelberg University.
- Hafsah Hachad is a medical doctor. As a nephrologist, she is interested in green nephrology. She also is a PhD student at the Université Technologique de Compiegne where she is preparing a thesis on the transition to more sustainable practices in dialysis care
- Prof. Dr. Rainer Sauerborn (born in 1952) is senior professor for climate change and health at the Heidelberg Institute of Global Health at the Heidelberg University Hospital.
- Anneliese Depoux is director of the Virchow-Villermé Center for Public Health Paris-Berlin at Paris Cité University. She holds a PhD in information and communication sciences where her research focuses mainly on the issue of media coverage of health crises related to environmental degradation, climate change and migration.
- Dr. med. Alina Herrmann is leading a research group on climate-smart health systems at the Heidelberg Institute of Global Health and is part of the lead team of research at the Institute of General Medicine at Cologne University. Her main research interest is the implementation of climate change adaptation and mitigation in the health sector.

## Competing interests

Alina Herrmann is member of the German Climate Change and Health Alliance (KLUG e.V.) and spokesperson of the Section on Climate Change and Health at the German Society for General and Familiy Medicine (DEGAM). She does not receive payments from these organizations. The other authors declare that they have no competing interests.

## Supplementary Material

Supplementary material
