# Supplementary material for: Integrating climate change and health topics into the medical curriculum – a quantitative needs assessment of medical students at Heidelberg University in Germany
Source: GMS J Med Educ. 2023 May 15;40(3):Doc36. doi: 10.3205/zma001618 (PMC10291352; doi:10.3205/zma001618)
Supplement: Supplementary material [file JME-40-36-s-001.pdf]

Attachment 1: Supplementary material

Table of contents

1 Survey 2

2 References for survey 12

3 Results of statistical analysis 14

3.1 Role perceptions 14

3.2 Knowledge 15

3.3 Learning needs and preference for educational strategies 21

3.4 Demographic characteristics 23

3.5 Correlations and regressions 25

Attachment 1 to Rybol L, Nieder J, Amelung D, Hachad H, Sauerbon R, Depoux A, Herrmann A. *Integrating climate change and health topics into the medical curriculum – a quantitative needs assessment of medical students at Heidelberg University in Germany.* GMS J Med Educ. 2023;40(3):Doc36. DOI: 10.3205/zma001618

1 Survey

This section contains the survey as it was administered online via Lime survey to the medical students at Heidelberg university. The green color in the answer options indicate the correct answers to the questions.

1 Sense of personal and professional role, identity and responsibility in the context of CC and health

In this section you will be asked about your personal and professional opinions, perceptions and attitudes about climate change and health. Please read the questions carefully and tick the answer that most fits your view. Please note there are no right or wrong answers.

R1 **Is climate change an issue you think and worry about a lot in your day-to-day life?**

- ◻ Not at all
- ◻ A little
- ◻ Moderately
- ◻ Quite a bit
- ◻ very often

Newly developed

R2 **In your personal life, do you engage in climate change action (e.g. political demonstrations, dietary behavior, less flying etc.)?**

- ◻ Not at all
- ◻ A little
- ◻ Moderately
- ◻ Quite a bit
- ◻ very much

Newly developed

R3 **How concerned are you about the impact climate change is having on your future patients' health?**

- ◻ Not at all
- ◻ A little
- ◻ Moderately
- ◻ Quite concerned
- ◻ Very concerned

Maibach et al. 2015; Ryan et al. 2020; adapted

R4 **Do you think that physicians have a responsibility to address climate change and health in their professional work setting?**

- ◻ Strongly disagree
- ◻ Disagree
- ◻ Neither agree nor disagree
- ◻ Agree
- ◻ Strongly agree

Sarfaty et al. 2014 ; adapted

R5 **Do you think that actions you take in your professional life as a physician can contribute effectively to mitigate climate change and adapt to its health impacts?**

- □ Strongly disagree
- □ Disagree
- □ Neither agree nor disagree
- □ Agree
- □ Strongly agree

Sarfaty et al. 2014 ; adapted

R6 **Do you feel like the medical training you have received so far, has imparted you enough skills to address climate change related health impacts and climate change mitigation in your future work as physician?**

- □ Strongly disagree
- □ Disagree
- □ Neither agree nor disagree
- □ Agree
- □ Strongly agree

Liao et al. 2019 ; adapted

---

2 Knowledge

In the following you will find some statements about climate change. Please read each statement carefully and indicate whether it is correct or incorrect. If you do not know the answer you can indicate that as well. Please only tick one answer per item.

W1 Climate change over the last 150 years is caused equally by human action and natural changes in the environment.

□ Correct □ Incorrect □ Don't know

Sarfaty et al. 2014 ; adapted

W2 It is very likely, that the increase of CO_2 and other greenhouse gases is the main cause of climate change.

□ Correct □ Incorrect □ Don't know

Newly developed
IPCC 2014

W3 One ton of emitted CO_2 is more harmful to the climate than one ton of emitted methane.

□ Correct □ Incorrect □ Don't know

Newly developed
IPCC 2014

W4 The ozone hole is one of the main causes of the greenhouse effect.

□ Correct □ Incorrect □ Don't know

Newly developed
IPCC 2014

W5 The 1-degree Celsius increase in global temperature that has been observed from 1850 to 2019 is detected uniformly across all latitudes from the equator to the poles.

□ Correct □ Incorrect □ Don’t know

Newly developed
IPCC 2014

In the following you will find some statements about the impacts of climate change on health. Please read each statement carefully and indicate whether it is correct or incorrect. If you do not know the answer you can indicate that as well. Please only tick one answer per item.

W6 During hot whether an increase in population mortality can be observed. Heat-related mortality starts to rise at different temperature thresholds depending on the geographic location (for example about 18°C in London and 27°C in Athens).

□ Correct □ Incorrect □ Don’t know

Newly developed
Baccini et al. 2008

W7 Vector-borne diseases such as Dengue-Fever are not affected by climate change because air temperature is a minor factor in the transmission cycle of such diseases.

□ Correct □ Incorrect □ Don’t know

Newly developed
Fouque et al. 2019

W8 The incidence of water-borne diseases such as infectious diarrhea, is likely to increase following severe storms or floods, depending on the status of water and hygiene infrastructure.

□ Correct □ Incorrect □ Don’t know

Newly developed
Cann et al. 2013

W9 Mortality due to respiratory diseases such as asthma and COPD is increased by air pollution but is not affected by rising temperatures.

□ Correct □ Incorrect □ Don’t know

Newly developed
Witt et al. 2015

W10 Extreme whether events are expected to become more frequent and severe as a consequence of climate change and lead to an increase in post-traumatic stress disorders, alcohol abuse and suicides.

□ Correct □ Incorrect □ Don’t know

Newly developed
Hayes et al. 2018

Attachment 1 to Rybol L, Nieder J, Amelung D, Hachad H, Sauerbon R, Depoux A, Herrmann A. *Integrating climate change and health topics into the medical curriculum – a quantitative needs assessment of medical students at Heidelberg University in Germany.* GMS J Med Educ. 2023;40(3):Doc36. DOI: 10.3205/zma001618

In the following you will find some statements about the vulnerability of populations towards the expected negative health impacts of climate change. Please read each statement carefully and indicate whether it is correct or incorrect. If you do not know the answer you can indicate that as well. Please only tick one answer per item.

W11 People with mental diseases are less vulnerable during a heatwave than those without mental diseases.

□ Correct □ Incorrect □ Don't know

Newly developed
Stivanello et al. 2020

W12 People with diabetes are more vulnerable to rising temperature since diabetes alters cooling mechanisms used by the body to avoid overheating.

□ Correct □ Incorrect □ Don't know

Newly developed
Charkoudian 2003

W13 People who are prone to allergies and asthma are a particularly vulnerable group because climate change affects the lengths of the pollen season and pollination patterns.

□ Correct □ Incorrect □ Don't know

Newly developed
Forsberg et al. 2012

W14 Elderly people are particularly vulnerable in heat waves, whereas outdoor workers or farmers are unlikely to suffer from the consequences of heat waves.

□ Correct □ Incorrect □ Don't know

Newly developed
Tawatsupa et al. 2012

W15 People living in coastal regions across all latitudes are highly vulnerable to the negative health effects of climate change due to rising sea levels and exposed geographic locations towards extreme weather events.

□ Correct □ Incorrect □ Don't know

Newly developed
Kulp et al. 2019

In the following you will find some statements about the adaptation of patient care to the health impacts of climate change. Please read each statement carefully and indicate whether it is correct or incorrect. If you do not know the answer you can indicate that as well. Please only tick one answer per item.

W16 Many anticholinergics, serotonin re-uptake inhibitors and tricyclic antidepressants impair central temperature regulation mechanisms and sweating.

□ Correct □ Incorrect □ Don't know

Newly developed
Westaway et al. 2015

W17 In a context of severe dehydration, physicians should increase the doses of drugs with renal clearance giving the risk of substantial decrease of glomerular filtration rate.

□ Correct □ Incorrect □ Don't know

Newly developed
ANSM 2014

W18 Ambient temperatures alter pharmacokinetics of oral medications but do not effect pharmacokinetics of trans- or subcutaneously applicated drugs.

□ Correct □ Incorrect □ Don't know

Newly developed
Hao et al. 2016

W19 Some antihypertensives drugs such ACE-Inhibitors and angiotensin receptor blockers can lessen the sensation of thirst and hasten acute renal failure in a period of hot weather.

□ Correct □ Incorrect □ Don't know

Newly developed
Westaway et al. 2015

W20 Weather Services across Europe, for instance the [insert French OR German weather service] issue locally tailored heat warnings before and during heat waves. They are embedded in broader heat health action plans, which i.a. involve health professionals to prevent heat deaths among vulnerable groups.

□ Correct □ Incorrect □ Don't know

Newly developed
Lowe et al. 2011

In the following you will find some statements about the associations of climate action and health. Please read each statement carefully and indicate whether it is correct or incorrect. If you do not know the answer you can indicate that as well. Please only tick one answer per item.

W21 Health co-benefits of actions mitigating climate change are defined as health gains, which result from actions mitigating climate change, such as climate-friendly policies or individual behavior.

□ Correct □ Incorrect □ Don't know

Newly developed
IPCC, 2018

W22 Health co-benefits from global climate action are not big enough to significantly reduce the disease burden of chronic diseases, such as cardiovascular or respiratory diseases.

□ Correct □ Incorrect □ Don't know

Newly developed
McMichael et al. 2011

Attachment 1 to Rybol L, Nieder J, Amelung D, Hachad H, Sauerbon R, Depoux A, Herrmann A. *Integrating climate change and health topics into the medical curriculum – a quantitative needs assessment of medical students at Heidelberg University in Germany.* GMS J Med Educ. 2023;40(3):Doc36. DOI: 10.3205/zma001618

W23 Savings in health care costs from reduced air pollution and associated lower disease burden have the chance to outweigh the investments needed for the transition to renewable energies in some countries, such as India and China.

□ Correct □ Incorrect □ Don't know

Newly developed
Markandya et al. 2018

W24 Active commuting (i.e. biking or walking) rather than car travel is an example of action that carries co-benefits because it decreases the risk for cardiovascular diseases and lowers CO_2 emissions.

□ Correct □ Incorrect □ Don't know

Newly developed
Woodcock et al. 2013

W25 Eating less red and processed meat has positive impacts on the reduction of greenhouse gas emissions but has no effect on reducing the risk of colorectal carcinoma.

□ Correct □ Incorrect □ Don't know

Newly developed
Aleksandrowicz et al. 2016

In the following you will find some statements about the associations of the health care system and climate change. Please read each statement carefully and indicate whether it is correct or incorrect. If you do not know the answer you can indicate that as well. Please only tick one answer per item.

W26 In European countries, greenhouse gas emissions from the health sector represent approximately 5% of all national greenhouse gas emissions.

□ Correct □ Incorrect □ Don't know

Newly developed
Karliner et al. 2020

W27 If the global health sector was a country, it would be one of the top five greenhouse gas emitters in the world.

□ Correct □ Incorrect □ Don't know

Newly developed
Karliner et al. 2020

W28 There is a strong but no absolute correlation between a country's health sector climate footprint and a country's health spending.

□ Correct □ Incorrect □ Don't know

Newly developed
Karliner et al. 2020

W29 Emissions from the health care supply chain meaning the production, transport and disposal of goods and services (such as pharmaceuticals, food and agricultural products or medical devices) make up about one third of health care's greenhouse gas emissions.

□ Correct □ Incorrect □ Don't know

Newly developed
Karliner et al. 2020

W30 The British National Health Service (NHS) has committed itself to severely reduce its greenhouse gas emissions and be climate neutral by 2045.

□ Correct □ Incorrect □ Don't know

Newly developed
NHS 2020

---

3 Learning needs and preference of educational strategies

In this section we would like to know your opinion about the integration of climate change and health into the medical curriculum. Please read the questions carefully and tick the answer that most fits your view. Please note there are no right or wrong answers

L1 In your opinion, should teaching about climate change and health be integrated into the medical curriculum?

- □ Strongly disagree
- □ Disagree
- □ Neither agree nor disagree
- □ Agree
- □ Strongly agree

Liao et al. 2019; adapted

If climate change is to be integrated in the medical curriculum, what key competency domains would you want to be taught?

L2 Health impacts of climate change (for instance setting non-communicable diseases, infectious diseases, mental health etc. into the context of climate change)

- □ Strongly disagree
- □ Disagree
- □ Neither agree nor disagree
- □ Agree
- □ Strongly agree

L3 Adaptation to climate change related health impacts (developing skills and building capacities in medical care for coping with health impacts of climate change on your future patients)

- □ Strongly disagree
- □ Disagree
- □ Neither agree nor disagree
- □ Agree
- □ Strongly agree

L4 Health co-benefits of climate action (learning about health gains going along with climate friendly behaviors and polices and building skills to integrate that knowledge in health counselling)

- ◻ Strongly disagree
- ◻ Disagree
- ◻ Neither agree nor disagree
- ◻ Agree
- ◻ Strongly agree

L5 Climate-friendly health sector (learning about sustainable health care solutions of practices, clinics and processes in medical care)

- ◻ Strongly disagree
- ◻ Disagree
- ◻ Neither agree nor disagree
- ◻ Agree
- ◻ Strongly agree

L6 Health advocacy and climate health policy (being taught about engaging in political commitment for systematic change)

- ◻ Strongly disagree
- ◻ Disagree
- ◻ Neither agree nor disagree
- ◻ Agree
- ◻ Strongly agree

Newly developed and adapted
Liao et al. 2019

If climate change is to be integrated in medical education systems, what training way(s) would you prefer?

L7 Offer a new optional independent course

- ◻ Strongly prefer
- ◻ Prefer
- ◻ Unsure
- ◻ Rather don’t prefer
- ◻ Don’t prefer

L8 Offer a new mandatory independent course

- ◻ Strongly prefer
- ◻ Prefer
- ◻ Unsure
- ◻ Rather don’t prefer
- ◻ Don’t prefer

L9 Integrate climate change into existing mandatory medical courses

- ◻ Strongly prefer
- ◻ Prefer
- ◻ Unsure
- ◻ Rather don't prefer
- ◻ Don't prefer

L10 Offer a specific specialty as doctor

- ◻ Strongly prefer
- ◻ Prefer
- ◻ Unsure
- ◻ Rather don't prefer
- ◻ Don't prefer

L11 Offer certified opportunities in continued medical education as doctors

- ◻ Strongly prefer
- ◻ Prefer
- ◻ Unsure
- ◻ Rather don't prefer
- ◻ Don't prefer

Liao et al. 2019 and Ryan et al. 2020; adapted

---

4 Demographic characteristics

In this last section, there are some questions about demographic characteristics and your current perceptions about your aspired career choice. Please answer these questions truthfully and according to your current judgement.

D1 What is your age?

- ◻ < 20
- ◻ 20-24
- ◻ 25-29
- ◻ > 30

Ryan et al. 2020; adapted

D2 What gender do you most associate with?

- ◻ Female
- ◻ Male
- ◻ Other
- ◻ Prefer not to answer

Ryan et al. 2020; adapted

D3 What semester are you currently in?

Newly developed

D4 What course are you currently taking?

Newly developed

D5 What specialty are you currently aspiring? Please only tick one answer.

- ◻ Allgemeinmedizin
- ◻ Anästhesiologie
- ◻ Arbeitsmedizin
- ◻ Augenheilkunde
- ◻ Chirurgie
- ◻ Gynäkologie
- ◻ HNO
- ◻ Dermatologie
- ◻ Hygiene und Umweltmedizin
- ◻ Innere Medizin
- ◻ Pädiatrie
- ◻ Klinische Chemie
- ◻ Mibi, Virologie, Infektio
- ◻ Neurologie
- ◻ Öffentliches Gesundheitswesen
- ◻ Pathologie
- ◻ Pharmakologie
- ◻ Psychiatrie/KJP
- ◻ Psychosomatik
- ◻ Radiologie
- ◻ Rechtsmedizin
- ◻ Strahlentherapie
- ◻ Urologie
- ◻ Sonstiges (specify):

Newly developed

D6 Where do you aspire a career in?

- ◻ a practice
- ◻ a hospital
- ◻ research
- ◻ industry

Sarfaty et al. 2014; adapted

D7 How would you describe your political leaning (voluntary answer)? Please only tick one answer.

- ◻ CDU/CSU
- ◻ SPD
- ◻ AfD
- ◻ FDP
- ◻ Linke
- ◻ Grüne
- ◻ Freie Wähler
- ◻ Die Partei
- ◻ ÖDP
- ◻ Piraten
- ◻ Volt
- ◻ Familie
- ◻ Prefer not to answer

Newly developed

2 References for survey

The references for the survey questions are listed in the following.

Aleksandrowicz L, Green R, Joy EJ, Smith P, Haines A. 2016. The Impacts of Dietary Change on Greenhouse Gas Emissions, Land Use, Water Use, and Health: A Systematic Review. PLoS One. 11(11):e0165797.

ANSM. 2014. Clarification regarding the correct use of medicinal products in the event of a heat wave. [accessed 2022 June 23]. https://archiveansm.integra.fr/var/ansm_site/storage/original/application/3304111144ff545812473 97b1ed94fed.pdf.

Baccini M, Biggeri A, Accetta G, Kosatsky T, Katsouyanni K, Analitis A, Anderson HR, Bisanti L, D'Ippoliti D, Danova J et al. 2008. Heat effects on mortality in 15 European cities. Epidemiology. 19(5):711-719.

Cann KF, Thomas DR, Salmon RL, Wyn-Jones AP, Kay D. 2013. Extreme water-related weather events and waterborne disease. Epidemiol Infect. 141(4):671-686.

Charkoudian N. 2003. Skin blood flow in adult human thermoregulation: how it works, when it does not, and why. Mayo Clin Proc. 78(5):603-612.

Forsberg B, Bråbäck L, Keune H, Kobernus M, Krayer von Krauss M, Yang A, Bartonova A. 2012. An expert assessment on climate change and health - with a European focus on lungs and allergies. Environ Health. 11 Suppl 1(Suppl 1):S4.

Fouque F, Reeder JC. 2019. Impact of past and on-going changes on climate and weather on vector-borne diseases transmission: a look at the evidence. Infectious Diseases of Poverty. 8(1):51.

Hao J, Ghosh P, Li SK, Newman B, Kasting GB, Raney SG. 2016. Heat effects on drug delivery across human skin. Expert Opin Drug Deliv. 13(5):755-768.

Hayes K, Blashki G, Wiseman J, Burke S, Reifels L. 2018. Climate change and mental health: risks, impacts and priority actions. Int J Ment Health Syst. 12:28.

IPCC. 2014. Climate Change 2014: Synthesis Report. Contribution of Working Groups I, II and III to the Fifth Assessment Report of the Intergovernmental Panel on Climate Change [Core Writing Team, R.K. Pachauri and L.A. Meyer (eds.)]. IPCC, Geneva, Switzerland, 151 pp.

IPCC. 2018. Annex I: Glossary [Matthews, J.B.R. (ed.)]. In: Global Warming of 1.5°C. An IPCC Special Report on the impacts of global warming of 1.5°C above pre-industrial levels and related global greenhouse gas emission pathways, in the context of strengthening the global response to the threat of climate change, sustainable development, and efforts to eradicate poverty [Masson-Delmotte, V., P. Zhai, H.-O. Pörtner, D. Roberts, J. Skea, P.R. Shukla, A. Pirani, W. Moufouma-Okia, C. Péan, R. Pidcock et al]. Cambridge University Press, Cambridge, UK and New York, NY, USA, pp. 541-562, doi:10.1017/9781009157940.008.

Karliner J, Slotterback S, Boyd R, Ashby B, Steele K, Wang J. 2020. Health care's climate footprint: the health sector contribution and opportunities for action. European Journal of Public Health. 30(Supplement_5):ckaa165.843.

Kulp S, Strauss B. 2019. New elevation data triple estimates of global vulnerability to sea-level rise and coastal flooding. Nature Communications. 10.

Liao W, Yang L, Zhong S, Hess JJ, Wang Q, Bao J, Huang C. 2019. Preparing the next generation of health professionals to tackle climate change: Are China's medical students ready? Environ Res. 168:270-277.

Lowe D, Ebi KL, Forsberg B. 2011. Heatwave early warning systems and adaptation advice to reduce human health consequences of heatwaves. Int J Environ Res Public Health. 8(12):4623-4648.

Maibach EW, Kreslake JM, Roser-Renouf C, Rosenthal S, Feinberg G, Leiserowitz AA. 2015. Do Americans Understand That Global Warming Is Harmful to Human Health? Evidence From a National Survey. Ann Glob Health. 81(3):396-409.

Markandya A, Sampedro J, Smith SJ, Van Dingenen R, Pizarro-Irizar C, Arto I, González-Eguino M. 2018. Health co-benefits from air pollution and mitigation costs of the Paris Agreement: a modelling study. The Lancet Planetary Health. 2(3):e126-e133.

McMichael AJ, Lindgren E. 2011. Climate change: present and future risks to health, and necessary responses. J Intern Med. 270(5):401-413.

NHS. 2020. Net Zero Expert Panel, Delivering a “Net Zero” National Health Service. [accessed 2020 June 23]. https://www.england.nhs.uk/greenernhs/wp-content/uploads/sites/51/2020/10/delivering-a-net-zero-national-health-service.pdf.

Ryan EC, Dubrow R, Sherman JD. 2020. Medical, nursing, and physician assistant student knowledge and attitudes toward climate change, pollution, and resource conservation in health care. BMC Med Educ. 20(1):200.

Sarfaty M, Mitchell M, Bloodhart B, Maibach EW. 2014. A survey of African American physicians on the health effects of climate change. Int J Environ Res Public Health. 11(12):12473-12485.

Stivanello E, Chierzi F, Marzaroli P, Zanella S, Miglio R, Biavati P, Perlangeli V, Berardi D, Fioritti A, Pandolfi P. 2020. Mental Health Disorders and Summer Temperature-Related Mortality: A Case Crossover Study. International Journal of Environmental Research and Public Health. 17(23).

Tawatsupa B, Lim LL, Kjellstrom T, Seubsman SA, Sleigh A. 2012. Association between occupational heat stress and kidney disease among 37,816 workers in the Thai Cohort Study (TCS). J Epidemiol. 22(3):251-260.

Westaway K, Frank O, Husband A, McClure A, Shute R, Edwards S, Curtis J, Rowett D. 2015. Medicines can affect thermoregulation and accentuate the risk of dehydration and heat-related illness during hot weather. J Clin Pharm Ther. 40(4):363-367.

Witt C, Schubert AJ, Jehn M, Holzgreve A, Liebers U, Endlicher W, Scherer D. 2015. The Effects of Climate Change on Patients With Chronic Lung Disease. A Systematic Literature Review. Dtsch Arztebl Int. 112(51-52):878-883.

Woodcock J, Givoni M, Morgan AS. 2013. Health impact modelling of active travel visions for England and Wales using an Integrated Transport and Health Impact Modelling Tool (ITHIM). PLoS One. 8(1):e51462.

3 Results of statistical analysis

3.1 Role perceptions

The descriptive results of the role perception section in the survey are listed in the following.

Table S1 Role perceptions results

						Likert scale responses [in %]				
**Question**	**Mean**	**Standard deviation**	**Median**	**Min.**	**Max.**	**1**	**2**	**3**	**4**	**5**
R1: Is climate change an issue you think and worry about a lot in your day-to-day life?	3.0824	0.7725	3	1	5	1.18	20	50.59	25.88	2.35
R2: In your personal life, do you engage in climate change action (e.g. political demonstrations, dietary behavior, less flying etc.)?	3.1471	0.9080	3	1	5	4.12	17.06	44.12	29.41	5.29
R3: How concerned are you about the impact climate change is having on your future patients' health?	3.2588	1.0676	3	1	5	6.47	16.47	32.94	32.94	11.18
R4: Do you think that physicians have a responsibility to address climate change and health in their professional work setting?	3.7529	0.9719	4	1	5	1.76	12.94	12.94	52.94	19.41
R5: Do you think that actions you take in your professional life as a physician can contribute effectively to mitigate climate change and adapt to its health impacts?	3.1412	1.0731	3	1	5	5.88	25.29	25.88	34.71	8.24

						Likert scale responses [in %]				
**Question**	**Mean**	**Standard deviation**	**Median**	**Min.**	**Max.**	**1**	**2**	**3**	**4**	**5**
R6: Do you feel like the medical training you have received so far, has imparted you enough skills to address climate change related health impacts and climate change mitigation in your future work as physician?	1.7412	0.8448	2	1	5	45.88	39.41	10	4.12	0.59
R1-5 Score	3.2765	0.6592	3.3	1.6	5					

3.2 Knowledge

The descriptive results of the knowledge section in the survey are listed in the following. Results for the question items individually, within sections and in total are presented below.

Table S2 Knowledge results

Question	Correctly answered [%]	Incorrectly answered [%]	Answered with do not know [%]	Mean of correctly answered questions in question group	Standard deviation of correctly answered questions in question group	Range of percentage share of correctly answered questions
**W1-5: Climate change**				3.3765	1.3542	53.53%-92.94%
W1 Climate change over the last 150 years is caused equally by human action and natural changes in the environment.						
	77.06	9.41	13.53			
W2 It is very likely, that the increase of CO_2 and other greenhouse gases is the main cause of climate change.						
	92.94	1.76	5.29			
W3 One ton of emitted CO_2 is more harmful to the climate than one ton of emitted methane.						
	57.65	2.94	39.41			
W4 The ozone hole is one of the main causes of the greenhouse effect.						
	56.47	18.24	25.29			
W5 The 1-degree Celsius increase in global temperature that has been observed from 1850 to 2019 is detected uniformly across all latitudes from the equator to the poles.						
	53.53	9.41	37.06			

Question	Correctly answered [%]	Incorrectly answered [%]	Answered with do not know [%]	Mean of correctly answered questions in question group	Standard deviation of correctly answered questions in question group	Range of percentage share of correctly answered questions
**W6-10: Climate change and health**				3.5824	1.0069	31.76%-100%
W6 During hot weather an increase in population mortality can be observed. Heat-related mortality starts to rise at different temperature thresholds depending on the geographic location (for example about 18°C in London and 27°C in Athens).						
	64.12	4.71	31.18			
W7 Vector-borne diseases such as Dengue-Fever are not affected by climate change because air temperature is a minor factor in the transmission cycle of such diseases.						
	82.35	1.18	16.47			
W8 The incidence of water-borne diseases such as infectious diarrhea, is likely to increase following severe storms or floods, depending on the status of water and hygiene infrastructure.						
	100	0	0			
W9 Mortality due to respiratory diseases such as asthma and COPD is increased by air pollution but is not affected by rising temperatures.						
	31.76	16.47	51.76			
W10 Extreme whether events are expected to become more frequent and severe as a consequence of climate change and lead to an increase in post-traumatic stress disorders, alcohol abuse and suicides.						
	80	3.53	16.47			
**W11-15: Climate change vulnerability**				3.7471	1.1150	44.12%-92.35%
W11 People with mental diseases are less vulnerable during a heatwave than those without mental diseases.						
	78.24	4.12	17.65			
W12 People with diabetes are more vulnerable to rising temperature since diabetes alters cooling mechanisms used by the body to avoid overheating.						
	44.12	7.06	48.82			
W13 People who are prone to allergies and asthma are a particularly vulnerable group because climate change affects the lengths of the pollen season and pollination patterns.						
	80	0	20			
W14 Elderly people are particularly vulnerable in heat waves, whereas outdoor workers or farmers are unlikely to suffer from the consequences of heat waves.						
	92.35	2.94	4.71			
W15 People living in coastal regions across all latitudes are highly vulnerable to the negative health effects of climate change due to rising sea levels and exposed geographic locations towards extreme weather events.						
	80	2.94	17.06			

Question	Correctly answered [%]	Incorrectly answered [%]	Answered with do not know [%]	Mean of correctly answered questions in question group	Standard deviation of correctly answered questions in question group	Range of percentage share of correctly answered questions
**W16-20: Climate change adaptation**				3.3118	1.2698	48.82%-83.53%
W16 Many anticholinergics, serotonin re-uptake inhibitors and tricyclic antidepressants impair central temperature regulation mechanisms and sweating.						
	74.71	0	25.29			
W17 In a context of severe dehydration, physicians should increase the doses of drugs with renal clearance giving the risk of substantial decrease of glomerular filtration rate.						
	48.82	7.65	43.53			
W18 Ambient temperatures alter pharmacokinetics of oral medications but do not effect pharmacokinetics of trans- or subcutaneously applicated drugs.						
	60	2.35	37.65			
W19 Some antihypertensives drugs such ACE-Inhibitors and angiotensin receptor blockers can lessen the sensation of thirst and hasten acute renal failure in a period of hot weather.						
	64.12	2.35	33.53			
W20 Weather Services across Europe, for instance the German weather service issue locally tailored heat warnings before and during heat waves. They are embedded in broader heat health action plans, which i.a. involve health professionals to prevent heat deaths among vulnerable groups.						
	83.53	1.18	15.29			
**W21-25: Climate action and health co-benefits**				2.7765	1.1397	11.18%-90.59%
W21 Health co-benefits of actions mitigating climate change are defined as health gains, which result from actions mitigating climate change, such as climate-friendly policies or individual behavior.						
	47.65	0.59	51.76			
W22 Health co-benefits from global climate action are not big enough to significantly reduce the disease burden of chronic diseases, such as cardiovascular or respiratory diseases.						
	11.18	27.06	61.76			
W23 Savings in health care costs from reduced air pollution and associated lower disease burden have the chance to outweigh the investments needed for the transition to renewable energies in some countries, such as India and China.						
	51.18	11.76	37.06			
W24 Active commuting (i.e. biking or walking) rather than car travel is an example of action that carries co-benefits because it decreases the risk for cardiovascular diseases and lowers CO_2 emissions.						
	90.59	0	9.41			
W25 Eating less red and processed meat has positive impacts on the reduction of greenhouse gas emissions but has no effect on reducing the risk of colorectal carcinoma.						
	77.06	3.53	19.41			

Attachment 1 to Rybol L, Nieder J, Amelung D, Hachad H, Sauerbon R, Depoux A, Herrmann A. *Integrating climate change and health topics into the medical curriculum – a quantitative needs assessment of medical students at Heidelberg University in Germany.* GMS J Med Educ. 2023;40(3):Doc36. DOI: 10.3205/zma001618

Question	Correctly answered [%]	Incorrectly answered [%]	Answered with do not know [%]	Mean of correctly answered questions in question group	Standard deviation of correctly answered questions in question group	Range of percentage share of correctly answered questions
**W26-30: Green healthcare system**				0.8353	1.0752	2.94%-31.18%
W26 In European countries, greenhouse gas emissions from the health sector represent approximately 5% of all national greenhouse gas emissions.						
	11.76	5.88	82.35			
W27 If the global health sector was a country, it would be one of the top five greenhouse gas emitters in the world.						
	14.12	3.53	82.35			
W28 There is a strong but no absolute correlation between a country's health sector climate footprint and a country's health spending.						
	31.18	5.88	62.94			
W29 Emissions from the health care supply chain meaning the production, transport and disposal of goods and services (such as pharmaceuticals, food and agricultural products or medical devices) make up about one third of health care's greenhouse gas emissions.						
	2.94	24.71	72.35			
W30 The British National Health Service (NHS) has committed itself to severely reduce its greenhouse gas emissions and be climate neutral by 2045.						
	23.53	1.18	75.29			

Table S3 Total knowledge score

	Mean	Standard deviation	Median	Min.	Max.
Correctly answered knowledge questions	17.6294	4.4878	18	3	29
Incorrectly answered knowledge questions	1.8235	1.6618	2	0	7
Knowledge questions answered with do not know	10.5471	5.0817	10	0	27

Figure S1 Item difficulty in knowledge question categories

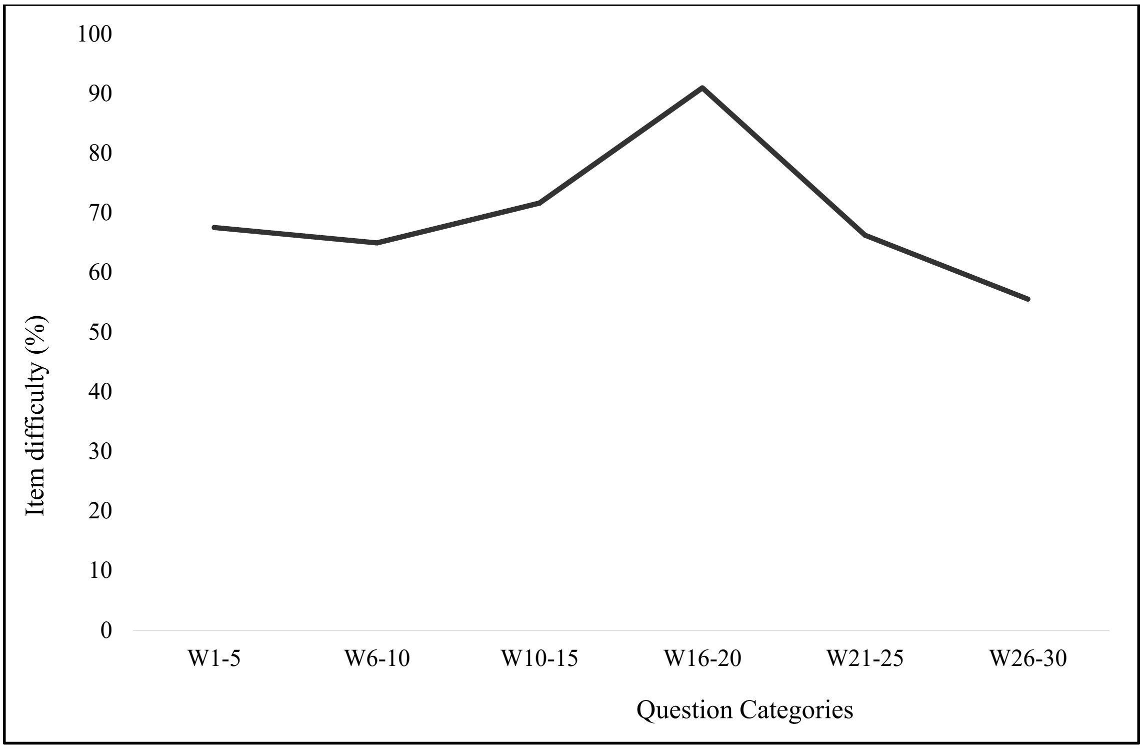

Figure S2 Item difficulty of knowledge questions

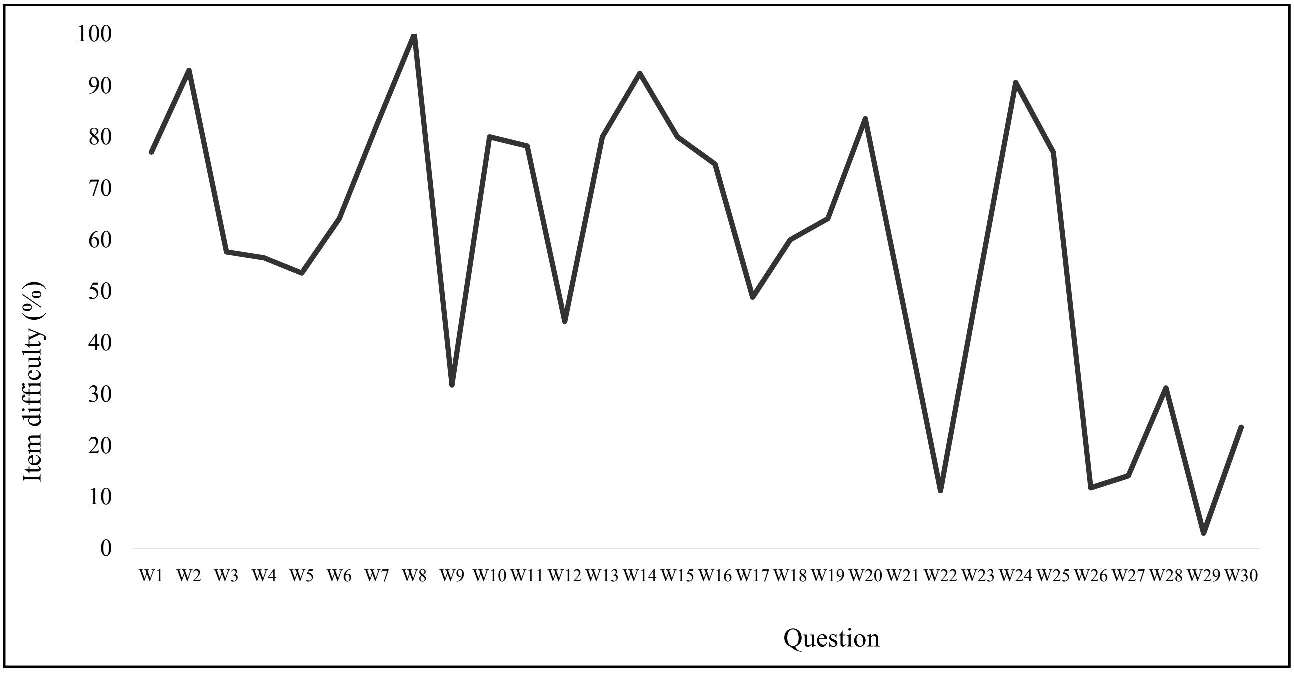

Table S4 Item selectivity of knowledge question

Knowledge question	Item selectivity (corrected item-scale correlation)
W1	0.305
W2	0.272
W3	0.339
W4	0.428
W5	0.410
W6	0.290
W7	0.379
W9	0.362
W10	0.343
W11	0.324
W12	0.329
W13	0.422
W14	0.381
W15	0.307
W16	0.215
W17	0.188
W18	0.322
W19	0.228
W20	0.240
W21	0.334
W22	0.324
W23	0.315
W24	0.354
W25	0.271
W26	0.431
W27	0.364
W28	0.442
W29	0.433
W30	0.323

3.3 Learning needs and preference for educational strategies

The descriptive results of readiness to learn and preference for educational strategies section in the survey are listed in the following.

Table S5 Learning needs and preference for educational strategies results

						Likert scale responses [in %]				
**Question**	**Mean**	**Standard deviation**	**Median**	**Min.**	**Max.**	**1**	**2**	**3**	**4**	**5**
L1: In your opinion, should teaching about climate change and health be integrated into the medical curriculum?	4.0706	1.0410	4	1	5	2.94	7.65	10	38.24	41.18
If climate change is to be integrated in the medical curriculum, what key competencies domains would you want to be taught?										
L2: Health impacts of climate change	4.4882	0.7070	5	2	5	0	2.35	5.29	33.53	58.82
L3: Adaptation to climate change related health impacts	4.2529	0.7999	4	1	5	1.18	1.76	10	44.71	42.35
L4: Health co-benefits of climate action	3.8588	0.9749	4	1	5	1.18	9.41	20	41.18	28.24
L5: Climate friendly health sector	3.9059	1.0946	4	1	5	3.53	9.41	15.29	36.47	35.29
L6: Health advocacy and climate health policy	3.6294	1.1349	4	1	5	4.12	15.88	17.06	38.82	24.12

						Likert scale responses [in %]				
**Question**	**Mean**	**Standard deviation**	**Median**	**Min.**	**Max.**	**1**	**2**	**3**	**4**	**5**
If climate change is to be integrated in medical education systems, what training ways would you prefer?										
L7: Offer a new optional independent course	3.5294	1.1315	4	1	5	6.47	14.71	15.29	46.47	17.06
L8: Offer a new mandatory independent course	2.8353	1.1802	3	1	5	14.12	27.06	29.41	20	9.41
L9: Integrate climate change into existing mandatory medical courses	3.8941	1.0550	4	1	5	4.12	6.47	17.06	40.59	31.76
L10: Offer a specific specialty as doctor	2.3059	1.0437	2	1	5	26.47	31.18	30	10	2.35
L11: Offer certified opportunities in continued medical education as doctors	4.1059	0.8774	4	1	5	2.35	3.53	8.82	51.76	33.53

3.4 Demographic characteristics

Demographic characteristics of survey participants are listed below. Note the differing N due to voluntary items age, gender, semester and political leaning.

Table S6 Demographic characteristics results

Demographic characteristic	N	Percentage (%)
Age (voluntary item)		
<20	2	1.20
20-24	127	76.05
25-29	24	14.37
>30	14	8.38
Gender (voluntary item)		
Female	95	56.21
Male	71	42.01
Diverse	3	1.78
Semester (voluntary item)		
5	99	58.58
6	26	15.38
7	17	10.06
8	4	2.37
9	15	8.88
10-14	8	4.73
Current course		
Propaedeutic semester	109	64.12
Internal medicine	39	22.94
Last clinical semester	22	12.94
Aspired specialty		
General medicine	13	7.65
Anesthesiology	14	8.24
Ophthalmology	5	2.94
Surgery	21	12.35
Dermatology	1	0.59
Ob/Gyn	17	10.00
Internal medicine	34	20.00
Microbiology, virology, infectiology	2	1.18
Neurology	10	5.88
Pathology	3	1.76
Psychiatry	9	5.29
Psychosomatic medicine	4	2.35
Pediatrics	14	8.24
Radiology	3	1.76

Attachment 1 to Rybol L, Nieder J, Amelung D, Hachad H, Sauerbon R, Depoux A, Herrmann A. *Integrating climate change and health topics into the medical curriculum – a quantitative needs assessment of medical students at Heidelberg University in Germany.* GMS J Med Educ. 2023;40(3):Doc36. DOI: 10.3205/zma001618

Urology	1	0.59
Nuclear medicine	1	0.59
Public health	1	0.59
Other	17	10.00
Aspired work setting		
Hospital	71	41.76
Practice	80	47.06
Research	16	9.41
Industry	3	1.76
Political leaning (voluntary item)		
AfD	1	0.70
CDU/CSU	2	1.40
Die Partei	4	2.80
FDP	18	12.59
Familie	2	1.40
Freie Wähler	1	0.70
Grüne	83	58.04
Linke	10	6.99
Piraten	2	1.40
SPD	12	8.39
Volt	7	4.90
ÖDP	1	0.70

3.5 Correlations and regressions

Cronbach's alpha, bivariate Pearson correlations and a summary of the regression statistics are listed below in the following section.

Table S7 Internal reliability of main scores measured with Cronbach's alpha

	**Cronbach's alpha**
Role perceptions (R1-5)	0.714
Knowledge (W1-30)	0.813
Readiness to learn (L1-6)	0.855

Table S8 Bivariate Pearson correlations between the dependent predictor variable readiness to learn and independent outcome variables knowledge and role perceptions

	**Knowledge**	**Role perceptions**
Readiness to learn	0.12360 (p=0.1083)	0.61840 (p<0.001)
Knowledge		0.28564 (p=0.0002)

Table S9 Summary of regression statistics. Predictor: learning needs

	**ΔR²**	**F**	**Unstandardized β**	**Standardized β**	**p-value**	**95% confidence intervals for β**		**VIF**
						**lower**	**upper**	
	0.459	17.858	13,443		<0.001	8.758	18.128	
**Age**			-0.628	-0.091	0.165	-1.517	0.261	1.093
**Gender**			-1.211	-0.147	0.027	-2.285	-0.137	1.123
**Semester**			0.440	0.182	0.006	0.131	0.749	1.072
**Aspired Setting**			0.218	0.037	0.572	-0.543	0.980	1.082
**Political leaning**			-0.047	-0.022	0.738	-0.325	0.231	1.134
**Role perception**			0.905	-0.112	0.108	-0.241	0.024	1.315
**Knowledge**			-0.109	0.669	< 0.001	0.714	1.097	1.227

Role Perception = R1-R5, Knowledge = total number of correct answers
